# Environmental pollutants as risk factors for neurodegenerative disorders: Alzheimer and Parkinson diseases

**DOI:** 10.3389/fncel.2015.00124

**Published:** 2015-04-10

**Authors:** Miguel Chin-Chan, Juliana Navarro-Yepes, Betzabet Quintanilla-Vega

**Affiliations:** Department of Toxicology, CINVESTAV-IPNMexico City, Mexico

**Keywords:** Alzheimer’s diseases, Parkinson’s disease, neurodegenerative disorders, beta-amyloid, tau protein, alpha-synuclein, metals, pesticides

## Abstract

Neurodegenerative diseases including Alzheimer (AD) and Parkinson (PD) have attracted attention in last decades due to their high incidence worldwide. The etiology of these diseases is still unclear; however the role of the environment as a putative risk factor has gained importance. More worryingly is the evidence that pre- and post-natal exposures to environmental factors predispose to the onset of neurodegenerative diseases in later life. Neurotoxic metals such as lead, mercury, aluminum, cadmium and arsenic, as well as some pesticides and metal-based nanoparticles have been involved in AD due to their ability to increase beta-amyloid (Aβ) peptide and the phosphorylation of Tau protein (P-Tau), causing senile/amyloid plaques and neurofibrillary tangles (NFTs) characteristic of AD. The exposure to lead, manganese, solvents and some pesticides has been related to hallmarks of PD such as mitochondrial dysfunction, alterations in metal homeostasis and aggregation of proteins such as α-synuclein (α-syn), which is a key constituent of Lewy bodies (LB), a crucial factor in PD pathogenesis. Common mechanisms of environmental pollutants to increase Aβ, P-Tau, α-syn and neuronal death have been reported, including the oxidative stress mainly involved in the increase of Aβ and α-syn, and the reduced activity/protein levels of Aβ degrading enzyme (IDE)s such as neprilysin or insulin IDE. In addition, epigenetic mechanisms by maternal nutrient supplementation and exposure to heavy metals and pesticides have been proposed to lead phenotypic diversity and susceptibility to neurodegenerative diseases. This review discusses data from epidemiological and experimental studies about the role of environmental factors in the development of idiopathic AD and PD, and their mechanisms of action.

## Introduction

Life expectancy has increased in last decades and health care improvements have contributed to people living longer. However, this has also contributed to increase the number of people with chronic disabling diseases such as Alzheimer (AD) and Parkinson (PD). Genesis of both neurodegenerative diseases has not been elucidated and several endogenous (genetic) and exogenous (environment) factors contribute to the onset and/or development of these illnesses, which highlights the necessity to expand the research on identifying the environmental risk factors that predispose to the development of these neurodegenerative diseases.

It is known that the etiology of neurodegenerative diseases is multifactorial, and there is evidence that potential external factors including lifestyle and chemical exposures are linked with the risk of the onset of these diseases. Since the vast majority of AD and PD cases are observed in elderly populations, yet the exposure to risk factors occurred years or decades before the diagnosis, the assessment of chronic exposures is difficult to perform in retrospective studies to associate them with the onset/development of the disease. Therefore more research for better definition of exposure, as well as for the identification of early specific biomarkers for the diagnosis of these diseases is needed. Attention is now focused on environmental factors that potentially damage the developing nervous system through epigenetic mechanisms, resulting in neurodegenerative diseases later in life. In this review we briefly examined the evidence of environmental etiologies related to two of the most common neurodegenerative diseases, AD and PD, from epidemiological as well as experimental studies.

### Alzheimer’s Disease

Alzheimer’s disease is the major form of dementia in elderly and possibly contributes to 60–70% of cases. It is a progressive, disabling and irreversible disease (Goedert and Spillantini, 2006). There are two recognized forms of AD. The first one is named familial or of early onset (EOAD), which is directly related to specific gene mutations in the amyloid precursor protein (APP) and presenilin (PSEN) 1 and 2 genes, both related to the amyloid-beta (Aβ) peptide synthesis (Piaceri et al., 2013). The EOAD begins at early age, less than 65 years, and only explains 5% of all cases. The second one, the late-onset or sporadic AD (LOAD) is the most common form of AD with 95% of all cases. This form of AD is not caused by punctual mutations, but some genetic risk factors have also been described such as polymorphisms in *ApoE* (encoding for apolipoprotein E), *SORL1* (encoding for neuronal receptor of ApoE), and *GSK3* (encoding for glycogen synthase kinase 3 beta) genes. The *ApoE* gene is the strongest genetic risk factor for LOAD, although it is not sufficient to explain the occurrence of the disease (Godfrey et al., 2003). Therefore, the etiology of LOAD remains unclear, and it is suggested that it has a multifactorial etiology.

Two hypotheses have been most studied for AD development. One is related to the overproduction of the Aβ peptide. According to this, neurofibrillary tangles (NFTs) result from the onset of amyloid deposits as Aβ plaques. While the second hypothesis suggests that the hyperphosphorylation of the Tau protein and its subsequent deposition as NFTs is the ultimate responsible for the disease. The amyloid cascade hypothesis establishes that Aβ aggregation initiates the brain damage leading to memory loss and to AD (Hardy and Higgins, 1992). Aβ is normally produced during aging, mediated by the proteolytic processing of the APP by the amyloidogenic enzymatic pathway. In this pathway, APP is processed by β- and γ-secretase complexes producing Aβ, soluble APPβ (sAPPβ) and the amyloid intracellular domain (AICD). Alternatively, APP can be processed by the non-amyloidogenic pathway leading to the production of AICD, sAPPα but not Aβ (Thinakaran and Koo, 2008). Thus Aβ increased levels in the brain of LOAD patients could be mediated by: (i) an increase in APP expression; (ii) an increase in the amyloidogenic pathway; or (iii) a reduction in the non-amyloidogenic pathway. It is stablished that the increase of a member of the β secretase complex, BACE1 (beta-site APP cleaving enzyme 1) produces high brain Aβ levels (Sun et al., 2012). On the other hand, the reduction on the activity of ADAM10 (a desintegrin and metalloproteinase domain-containing protein 10) could also lead to the overproduction of Aβ (Kojro and Fahrenholz, 2005). Furthermore, some mutations such as those in PSEN 1 or 2, a catalytic member of the γ-secretase complex, can also increase the production of Aβ (Piaceri et al., 2013).

Another mechanism to increase brain Aβ levels is through a reduction in the Aβ degradation. There are proteins collectively known as Aβ-degrading enzyme (IDE)s that have the ability to degrade Aβ, including insulin-like IDE, angiotensin-converting enzyme (ACE), endothelin-converting enzyme (ECE), plasmin, cathepsin B, aminopeptidase A, matrix metalloproteinase (MMP) 2 and 9, neprilysin (NEP, neutral endopeptidase) and others. These enzymes have been suggested as viable therapeutic targets for AD treatment (Nalivaeva et al., 2012). Finally, a reduced brain clearance of Aβ can be another pathway for the brain accumulation of Aβ. Some cholesterol transporters such as the low density lipoprotein receptor-related protein 1 (LRP1) are involved in the Aβ export from the brain to the cerebrospinal fluid (CSF). This receptor links the imbalance of cholesterol homeostasis with AD pathogenesis (Zlokovic et al., 2010).

On the other hand, aggregates of the microtubule (MT)-associated protein Tau observed in cell bodies and apical dendrites as NFTs cause neurofibrillary lesions associated with AD. Tau is a phosphoprotein mainly localized in the axon of neurons for the stabilization of MTs; it contains a high number of serine and threonine residues, and is therefore a substrate of many kinases (Goedert et al., 1988). The abnormal aggregation of Tau into insoluble paired helical filaments (PHFs), which are the major component of NFTs found in cell bodies and apical dendrites of neurons are lesions associated with AD (Friedhoff et al., 2000). Under pathological conditions, Tau is hyperphosphorylated at “pathological” sites leading to MTs depolymerization, axonal transport disruption and aggregation (Götz, 2001). It has been proposed that repeat domains (RD) of the MT-binding domain (MBD) in the C-terminal structure of Tau can rapidly form PHFs compared with the complete protein, suggesting that RDs are indispensable for its aggregation (Wille et al., 1992), and for Tau filament formation (Tokimasa et al., 2005).

There is no cure for AD, and therapeutic treatments are basically to ameliorate the symptoms. Therefore, an early and opportune diagnosis is indispensable to slow the progression of the disease. Currently, the determination of Tau and Aβ levels in blood and CSF are broadly used for the diagnosis of AD, and several medical tools are also used to confirm the diagnosis including the medical history, mental status tests, and evaluations of the brain structure and function with neuroimaging techniques (Lewczuk et al., 2014). However, these biomarkers are not sensitive nor specific for AD. Interestingly, an emerging body of evidence suggests that micro RNAs (miRNAs, small non-coding RNAs involved in the post-transcriptional regulation of gene expression) could be putative biomarkers for detecting neurodegeneration Thus, recent reviews have shown that some miRNAs are differentially associated with AD by modulating the expression of important genes involved in Aβ production (e.g., *BACE1*) or inflammation (Goodall et al., 2013; Van den Hove et al., 2014).

#### Aβ Homeostasis as a Target of Environmental Factors

**Figure 1:**
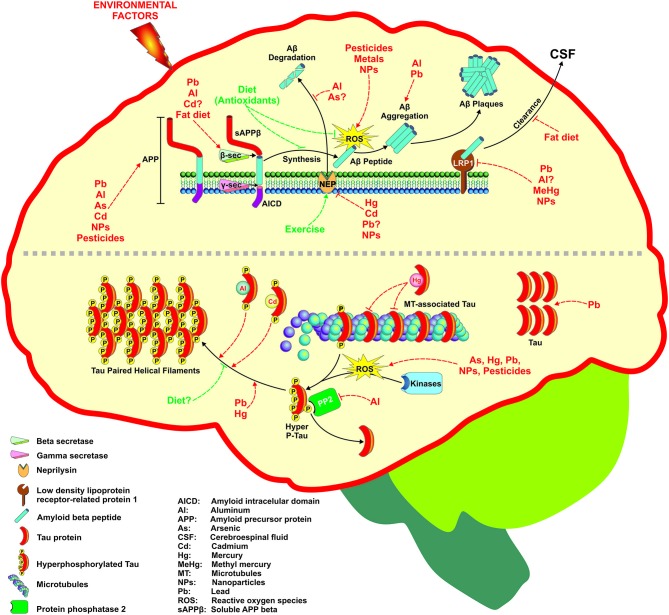
**Environmental factors associated with Alzheimer’s disease (AD) development through different mechanisms**. Several factors including metals, pesticides, nanoparticles, and diet can affect the two targets of AD such as Aβ generation and Tau phosphorylation. The figure depicts the molecular targets than can be modified at different levels following the amyloid hypothesis that ends in Aβ senile plaques formation (upper part) or the hyperphosphorylation of Tau protein and its subsequent deposition as neurofibrillary tangles (NFTs) (lower part). For more detail see the text.

Environmental factors such as diet (fat-rich), heavy metals, biogenic metals and pesticides have been involved in AD development due to their ability to disrupt metabolic pathways involved in the homeostasis of Aβ. In addition, factors such as lifestyle (antioxidants and exercise) can prevent AD development (Figure 1). Many of these environmental factors are oxidative agents acting through different mechanisms as discussed later. The brain is particularly vulnerable to oxidative stress do to its high glucose-based metabolic rate, low levels of antioxidants, high levels of polyunsaturated fatty acids, and high enzymatic activities related to transition metals that catalyze the formation of free radicals (Halliwell et al., 1992). In addition, micromolar concentrations of Aβ induce the formation of H_2_O_2_ in culture cells leading to neurotoxicity, and the presence of some antioxidant enzymes prevents the toxicity of the peptide (Butterfield et al., 2001). The mechanism by which Aβ generates free radicals is not known, and other endogenous factors also generate reactive oxygen species (ROS) in AD. For instance, the ion Fe^3+^, which is at high concentrations in NFTs and Aβ-aggregates, catalyzes the formation of reactive species such as H_2_O_2_, as well as advanced glycation end products (AGE) that are related to neurodegeneration (Smith et al., 1997b). On the other hand, activated microglia that surrounds the senile plaques is a source of NO and O_2_ (Cras et al., 1990), which can react to form the peroxinitrite radical (ONOO-) (Smith et al., 1997a). Likewise, inflammation has gained importance in AD pathogenesis (Tuppo and Arias, 2005). The central nervous system is considered a privileged site with its own immune system and microglia and astrocytes are the principal cells involved in the inflammatory response. It is accepted the microglial chemotaxis of Aβ and the phagocytosis of amyloid fibrils, effects that produce an increase in the secretion of pro-inflammatory cytokines and ROS, which in consequence produces neuronal loss (Rogers and Lue, 2001). In agreement, astrocytes are also recruited into amyloid plaques for Aβ degradation (Wyss-Coray et al., 2003), and it is possible that the activation of microglia and astrocytes is a consequence of Aβ aggregation. The role of inflammatory processes in AD is supported by the use of non-steroidal anti-inflammatory drugs (NSAIDs) to reduce the Aβ levels (Weggen et al., 2001), and the risk of AD (Etminan et al., 2003).

##### Metals

Lead (Pb) is a heavy metal well known by its neurological toxic effects, although a direct association with AD development has not been reported. Pb affects cognitive abilities, intelligence, memory, speed processing and motor functions in children (Mason et al., 2014), while studies in elderly are limited. A cohort study reported that bone Pb levels were associated with poor cognitive performance scores in old workers, suggesting that past Pb exposure can contribute to late cognitive deterioration (Dorsey et al., 2006). However, a recent study reported no association between serum Pb levels and AD (Park et al., 2014). Despite the few epidemiological studies relating Pb exposure with AD, the evidence is more solid in experimental studies. The influence of Pb in AD was initially suggested from results in rats early exposed (from postnatal day 1–20) or late exposed (at 18–20 months of age) to Pb (200 ppm, drinking water). Results showed an increase in the *APP* mRNA expression late in life after the neonatal exposure, but not in rats exposed as adults (Basha et al., 2005). Similarly, a study performed in non-human primates (*Macaca fascicularis*) exposed to Pb (1.5 mg/Kg/day, from birth to 400 days) showed that monkeys exposed at a young age had an increased number of amyloid plaques late in life (at 23 years old) (Wu et al., 2008). The increased Aβ levels appear to be mediated by an augmented expression of *APP* and *BACE1* (Wu et al., 2008). These effects were also observed in differentiated SH-SY5Y cells incubated with Pb (5–100 µM/48 h) and analyzed 6 days later (Bihaqi and Zawia, 2012). Another study performed in differentiated SH-SY5Y cells showed an increase in Aβ secretion and *APP* expression, as well as reduced expression and protein levels of NEP (an Aβ IDE), suggesting that Pb can target both the synthesis and degradation of Aβ (Huang et al., 2011). However, in a recent work conducted in our laboratory, Pb did not show changes in *NEP* expression in differentiated SH-SY5Y cells exposed to 50 µM Pb, but an increase in APP levels (Chin-Chan et al., 2015). Another mechanism by which Pb increases Aβ levels is by reducing the brain Aβ clearance. A recent study showed that acute Pb exposure (27 mg/Kg, i.p.) to APP transgenic mice (V717F) reduced the expression of *LRP1*, resulting in the accumulation of Aβ in the hippocampus and cortex of treated mice (Gu et al., 2011). Studies from this group support that Pb can disrupt the brain export of Aβ leading to its accumulation and plaques formation (Behl et al., 2009, 2010).

Exposure to Pb during development is a good example of an environmental contaminant as a risk factor to promote neurodegenerative diseases such as AD, supporting the hypothesis that many adult diseases have a fetal origin (FeBAD) (Basha et al., 2005). The group of Zawia has extensively worked on latent responses to prenatal and early postnatal exposures to Pb. Authors exposed male neonatal rats to Pb (200 ppm, drinking water) from postnatal day 1–20, or to aging animals (18–20 months of age), and observed an increase in the *APP* mRNA expression as well as in the activity of the transcription factor Sp1 (one of the regulators of *APP*) in the cortex of neonates, and after 20 months of Pb exposure had ceased. They observed a concomitant increase in Aβ levels in old animals exposed to Pb at birth. Interestingly, APP and Aβ protein levels did not respond to Pb exposure at old age (Basha et al., 2005). Similarly, a study conducted in cynomolgus monkeys exposed to Pb (1.5 mg/Kg/day, via infant formula) from birth to 400 days of age, and terminated 23 years later showed increased mRNA levels of *APP* and *Sp1* in the frontal cortex compared with control animals, and high levels of the biomarker of oxidative DNA damage, 8-oxo-dG, suggesting an oxidative mechanism (Wu et al., 2008). Aβ1–42 and Aβ-1–40 levels in aged monkeys were also increased, as well as the intracellular Aβ staining and dense-plaques compared with age-matched controls (Wu et al., 2008). In addition, a study reported that gestational exposure to Pb (0.1, 0.5 and 1%, drinking water) in mice led to increased brain levels of Aβ and worst spatial memory performance, as well as increased levels of pro-inflammatory agents such as interleukin-1 (IL-1) and tumor necrosis factor alpha (TNF-α; Li et al., 2014a).

Mercury (Hg) is a heavy metal with a high potential to cause neurotoxicity. Early studies about Minamata and Iraq disasters led us to understand the neurotoxicity of this metal. It is widely accepted that Hg disrupts the brain development and produces cognitive and motor disabilities (Johansson et al., 2007), and in adults, Hg exposure produces memory loss and cognitive alterations (Wojcik et al., 2006; Chang et al., 2008). An early study suggested a link between Hg exposure and AD. Authors reported increased levels of Hg (in microsomes) and bromide (in the whole brain), and reduced levels of rubidium (in the whole brain, microsomes, and nuclei), selenium (Se; in microsomes) and zinc (Zn; in nuclei) in AD brains compared with controls (Wenstrup et al., 1990). A subsequent work reported more than a 2-fold increase in blood Hg levels in AD patients (*n* = 33) compared with a control group (*n* = 65), as well as a positive correlation between blood Hg concentration and CSF levels of Aβ (*n* = 15, Pearson’s correlation *r* = 0.7440, *p* = 0.0015) (Hock et al., 1998). More recently, several metals, including manganese (Mn), nickel, cadmium (Cd), Pb and Hg were determined in plasma and CSF of AD patients (*n* = 173) and healthy controls (*n* = 54), however only plasma Mn and Hg concentrations were significantly higher in AD patients (Gerhardsson et al., 2008). On the other hand, ApoE4 is a risk factor for AD, probably because this protein does not have sulfhydryl (SH) groups to scavenge heavy metals like Hg, whereas ApoE2 has four SH groups and then the ability to reduce the metal toxicity in the brain (Mutter et al., 2004). Therefore, *ApoEε2* is considered a protective genotype for AD development (Suri et al., 2013). Similarly, Godfrey et al. (2003) observed a shift toward the risky *ApoEε4* allele in patients with presumptive Hg-related neuropsychiatric symptoms with an elevated Hg body burden (*n* = 400; Godfrey et al., 2003).

The ability of Hg to increase Aβ levels has been studied *in vitro* and *in vivo*, and the suggested mechanisms include an increased production, a reduced degradation and/or a diminished brain clearance of the peptide. Olivieri et al. (2000) reported an increased secretion of both Aβ-40 and Aβ-42 when neuroblastoma cells were exposed to 50 µg/dL of inorganic Hg concomitant with ROS overproduction (Olivieri et al., 2000), and a study conducted in aggregating brain-cell cultures of fetal rat telencephalon showed that MeHg (non-cytotoxic concentrations/10–50 days) produced increased APP levels accompanied by ROS production and glia activation (Monnet-Tschudi et al., 2006). Rat pheochromocytoma (PC12) cells exposed to both inorganic and organic (MeHg) Hg (10–1000 nM) also showed a dose-dependent overproduction of Aβ-40 probably by an increase in APP levels as well as to a reduction in Aβ degradation by NEP (Song and Choi, 2013). However, an increase in Aβ-42 levels was observed in differentiated SH-SY5Y cells exposed to Hg (10 and 20 µM) but not in the *APP* expression, and rather a reduced activity of the Aβ-degrading enzyme, NEP was observed (Chin-Chan et al., 2015). Negative effects of Hg on Aβ aggregation have also been published, for example, Atwood et al. (1998) studied the role of the pH and the ability of various metals to aggregate Aβ; Zn, Cu and Fe showed the highest potential on Aβ aggregation, and Hg did not show an important effect (Atwood et al., 1998). Regarding *in vivo* studies, oral administration of 20–2000 µg/Kg/day/4 weeks of MeHg produced a dose-dependent increase in Aβ-42 in the hippocampus of male rats, but not significant changes in APP or NEP protein levels (Kim et al., 2014). Interestingly, authors observed a reduced brain expression of the *LRP1* receptor, which was positively correlated with increased Aβ levels in the hippocampus and reduced levels in the CSF, suggesting a reduced clearance of the pathogenic peptide from the brain (Kim et al., 2014).

Inorganic arsenic (As) is a known neurotoxic metalloid with adverse effects in both the neurodevelopment and cognitive function (Tyler and Allan, 2014), although its effects in elderly have been less studied. There are few papers evaluating the role of As exposure as a risk factor for AD development. A study conducted in rural-dwelling adults and elders in Texas, US (FRONTIER project) reported that long-term exposure to low As levels (3–15 µg/L As in water) correlated (after adjustment by confounders, including the *ApoEε4* presence) with a poor score of cognitive abilities and memory, which reflects the earliest manifestations of AD (O’Bryant et al., 2011). Similarly, a positive correlation was observed between serum As levels and the cognitive ability in AD patients from Hong Kong (*n* = 44, Pearson’s correlation coefficient *r* = 0.55, *p* < 0.0001) compared with matched controls (*n* = 41), lower serum Zn concentrations were observed in AD patients as well (Baum et al., 2010). On the other hand, the study conducted in several European countries showed a higher prevalence of AD and other dementias in those countries with As levels in topsoils about 18 ppm such as Italy, Switzerland, Spain, France, Belgium and Norway, compared with countries with lower As levels (in the range of 9 ppm), including Luxemburg, Denmark, Finland, UK and Nertherlands (Dani, 2010). In experimental studies, the administration of inorganic As (20 mg/L, drinking water during the gestation and early postnatal life) to mice produced a significant loss of spatial memory (Ramos-Chávez et al., 2015).

A plausible mechanism for the cognitive and memory alterations induced by As exposure is by alterating the amyloid pathway. Zarazúa et al. (2011) reported that cholinergic SN56.B5.G4 cells incubated with sodium arsenite or the organic form dimethylarsinic acid (DMA) (5–10 µM/12–24 h) showed an increase in APP and sAPPβ levels, and consequently an increase in Aβ only with DMA. Similar effects were observed in neurons from Tg2576 mice (a murine model that overexpresses a mutant form of APP most used in AD). They suggest that DMA-induced effects may be due by an increased Aβ anabolism (enhanced *APP* expression), although authors did not discard an alteration in the Aβ degradation pathway (Zarazúa et al., 2011). The mechanism by which As causes Aβ overproduction has not been determined, but As exposure has been associated with brain inflammatory responses and oxidative stress, which is in agreement with the inflammatory and oxidative hypotheses of AD (Gong and O’Bryant, 2010).

Cadmium is another toxic heavy metal associated with neurological alterations including memory loss and mental retardation (Wang and Du, 2013). An early study observed higher plasma levels of various metals including Cd, aluminum (Al), As, and Se in AD patients (*n* = 24) compared with healthy volunteers (*n* = 28) (Basun et al., 1991). Also, the liver from autopsied AD patients (*n* = 17) had significant higher Cd levels compared with age- and sex-matched control subjects (*n* = 17) (Lui et al., 1990). However, Gerhardsson et al. (2008) did not observe significant differences in Cd concentrations in plasma or CSF in patients with AD (*n* = 173) compared with healthy control subjects (*n* = 54) (Gerhardsson et al., 2008). There is evidence linking Cd exposure with Aβ overproduction. Li et al. (2012) observed cognitive alterations accompanied by an increased production of Aβ-42 and increased size and number of senile plaques in the cerebral cortex and hippocampus from APP/PSEN1 transgenic mice treated with Cd (2.5 mg/Kg/4 days, drinking water). These effects were attributed to a reduced expression of ADAM10, sAPPα and NEP proteins, suggesting that the non-amyloidogenic pathway as well as Aβ degradation are target of Cd exposure (Li et al., 2012). Additionally, authors reported an increase in free-Zn levels, suggesting that Cd displaces Zn from its native enzymes, including NEP.

Recently, Ashok et al. (2015) investigated the role of the exposure to individual metals (As, Pb and Cd) and their combination in the AD-amyloid pathway in male rats exposed from gestational day 5 to postnatal day 80 through drinking water. They reported that metals activated the synthesis of Aβ in the frontal cortex and/or hippocampus, mediated by an increase in APP, and in APP-processing enzymes such as beta secretases BACE1 and PSEN at postnatal days 24 (post-weaning) and 90 (adulthood). Pb was the most potent metal to induce Aβ, followed by Cd, and As had the smallest effect, however all did increase the APP production. Interestingly, they demonstrated a synergic effect of metals mainly due to As, the exposure to these three cations produced a dramatic increase in Aβ, PSEN1, BACE1 and APP, suggesting an enhanced amyloidogenic processing (Ashok et al., 2015). Authors also observed (Ashok et al., 2015) increased levels of malondialdehyde (MDA), reduced activity of antioxidant enzymes, and the induction of 1L-1α and IL-1β in the frontal cortex and hippocampus of rats exposed to As + Pb + Cd mixture. Authors suggest that ROS-induced IL-1 overproduction was responsible for the *APP* expression. This is supported by the fact that the 5’ÚTR region of the mRNA of *APP* has a responsive element for IL-1 (Rogers et al., 1999; Ashok et al., 2015).

Aluminum is a neurotoxic element involved in the etiology of neurodegenerative disorders such as AD; however, there is no consistent evidence. The incident of Al pollution in Cornwall, UK (1998) gave evidence of Al potential neurotoxicity. Similar brain pathological characteristic found in AD patients were observed in subjects exposed to Al in this region (Exley and Esiri, 2006), as well as alterations in brain functions (Altmann et al., 1999). A recent study conducted in China reported a marginal positive association between Al levels in soil and the mortality caused by AD (Shen et al., 2014); while other studies reported no association. Experimental evidence appears to be more consistent. Chronic oral Al administration in rats (20 g/day in the food/twice weekly from 6 months of age to the end of their lives) increases the Aβ production by raising the levels of APP in hippocampal and cortical tissues (brain regions important for the memory process) (Walton and Wang, 2009). Cultured rat cortical neurons exposed to Al (50 µM/48 days) showed an accumulation of Aβ; furthermore, Al induced conformational changes in Aβ and enhanced its aggregation forming fibrillar deposits on the surface of cultured neurons. The aggregated Aβ was dissolved by the addition of desferroxamine, a chelator of Al (Exley et al., 1993; Kawahara et al., 2001). The administration of Al plus D-galactose (an animal model for AD) produced the impairment of memory and increased the production of Aβ in the cortex and hippocampus. Additionally, an augmented expression of *BACE1* and a reduction of *NEP* were observed in this co-treatment (Luo et al., 2009). Another study showed that Al reduced the Aβ degradation by decreasing the activity of cathepsin B, suggesting the activation of the amyloidogenic pathway and a reduction of the catabolism of Aβ (Sakamoto et al., 2006). In addition, a reduced expression of *LRP1* was also observed in these mice administered with Al plus D-galactose, indicating a possible reduction of the clearance of Aβ as well (Luo et al., 2009). Transgenic mice (Tg2576) fed with Al (2 mg/Kg, in the diet/9 months) showed an increased production of Aβ and proteins involved in its anabolism; the accumulation of amyloid plaques were reversed by the treatment with vitamin E, suggesting the contribution of Al-induced oxidative mechanism (Praticò et al., 2002).

As mentioned earlier, miRNA can be biomarkers of early diagnosis of AD, however few studies have reported the involvement of pollutants in the miRNAs homeostasis (Ray et al., 2014). Interestingly, Tg2576 mice exposed to Al (2 mg/Kg/9 months, through the diet) showed an increased expression of miRNAs (e.g., miR146a and miR125b) involved in a pro-inflammatory response similar to that observed in AD brains (Bhattacharjee et al., 2014; Zhao et al., 2014), and treatment of primary human astroglial (HAG) cells with 100 nM of Al + Fe increased the expression of NFκB-induced miR-125b and miR-146a (Pogue et al., 2011); these miRNAs are reported in AD pathology (Lukiw, 2012). Further studies are necessary to look for a possible relation between xenobiotic exposures and the deregulation of miRNA expression involved in neurodegeneration.

##### Pesticides

The association between chronic pesticide exposure and the prevalence of dementias, including AD has not been as well studied as with other environmental risk factors, and results are often inconsistent. This is mainly because the difficulty on getting adequate data on the levels of exposure of individual pesticides, which is often indirectly evaluated by structure questionnaires (exposure index). Some of the studies with positive associations include the one performed in the agricultural Cache County, Utah, US in about 3000 occupationally pesticides-exposed participants who were followed-up for 3, 7 and 10 years. The hazard ratio for developing AD was slightly higher for organophosphate (OP) pesticides exposure (HR = 1.53, 95% CI, 1.05–2.23) than to organochlorines (OCl) (HR = 1.49, 95% CI, 0.99–2.24), after adjusting for some variables, including *ApoE* genotype (Hayden et al., 2010). Similarly, a recent case-control study observed a 3.8-fold increase in the OCl metabolite DDE in serum from AD patients (*n* = 79) compared with control participants (*n* = 86); in addition, authors reported that the highest tertile of DDE levels was associated with an increased risk for AD development (odds ratio-OR = 4.18, 95% CI, 2.54–5.82), and carriers of an *ApoEε4* allele may be more susceptible (Richardson et al., 2014). Baldi et al. (2003) evaluated the association between lifelong cumulative exposure to pesticides and neurodegenerative diseases in a subsample from a cohort of elderly people (aged 65 years or older) (PAQUID study) in southwestern France. Authors analyzed 96 incident cases of AD (71 women and 25 men) in a 5-year follow-up approach, and observed a significant association between AD and occupational exposure to pesticides in men with a relative risk of 2.4 (95% CI, 1.0–5.6) after adjusting by education and smoking. Results were not significant in women (Baldi et al., 2003).

The role of pesticides in alterations observed in cognitive functions and AD has been suggested based on epidemiological studies, but the mechanisms have been poorly explored. *In vitro* studies performed in differentiated SH-SY5Y cells incubated with OCl pesticides such as DDE and its parent compound DDT (1 µM/48 h) showed increased APP protein levels, although authors did not evaluate Aβ levels (Richardson et al., 2014). A recent study reported that DDT augmented Aβ levels by increasing APP and BACE1 levels in human neuroglioma H4-AβPPswe cells, as well as by reducing the clearance and degradation of the peptide by targeting the Aβ-degrading enzyme, IDE and the ATP-binding cassette transporter A1 (ABC1; Li et al., 2015). Regarding *in vivo* data, chlorpyrifos (CPF), an OP insecticide associated with cognitive impairment, oxidative stress and neuronal damage caused a significant increase in Aβ levels in the cortex and hippocampus, as well as increased memory loss and reduced motor activity in Tg2576 mice 6 months after an acute subcutaneous administration of 50 mg/Kg of CPF (Salazar et al., 2011). However, Peris-Sampedro et al. (2014) did not find increased Aβ levels neither significant changes in memory acquisition in Tg2576 mice treated with CPF (25 mg/Kg/twice weekly/4 weeks, intragastric) and analyzed 6 months later (Peris-Sampedro et al., 2014). More studies are needed to better understand the mechanisms by which OCl, OP and other insecticides are linked to AD.

Paraquat (PQ), is a common used herbicide that has been suggested to be involved in AD development. A recent study showed that treatment of wild type and APP transgenic (Tg2576) mice with PQ (10 mg/Kg/twice a week/3 weeks) produced a significant increase in Aβ levels in transgenic mice that was associated with mitochondrial oxidative damage in cerebral cortex leading to the impairment of learning and memory. Interestingly, the overexpression of peroxiredoxin 3, a mitochondrial antioxidant defense enzyme produced an improvement in cognitive functions and a significant reduction in Aβ levels in APP transgenic mice exposed to PQ (Chen et al., 2012a), suggesting that pro-oxidant xenobiotics like PQ can contribute to AD.

##### Nanoparticles

As the synthesis of NPs for different applications, including drug delivery strategies in the treatment of AD is growing, it is necessary to study the potential toxic effects on proteins related to AD development.

There are not epidemiological studies associating the exposure to NPs with AD. However, there is increasing experimental evidence suggesting the potential role of NPs in brain damage. A recent study reported that nasal administration of TiO_2_-NPs (2.5–10 mg/Kg/90 days) to mice caused neuronal death in the hippocampus, oxidative stress and gliosis, and microarray analysis revealed a decline of genes associated with memory and cognition (Ze et al., 2014). Similarly, rats exposed to CuO-NPs (0.5 mg/Kg/day/14 days, i.p.) showed worst spatial cognition and a reduction in electrophysiological endpoints such as long-term potentiation, which matched with augmented levels of ROS and lipid peroxidation products (MDA and 4-hydroxinonenal-HNE), and reduced levels of antioxidants enzymes (An et al., 2012). Studies of NPs of Al, Cu and Ag administered at different doses and routes in rats and mice showed that they produce brain alterations such as motor, sensory and cognitive deteriorations (Sharma et al., 2009; Sharma and Sharma, 2012). However a recent study did not observe memory loss in adult mice administered with Ag-NPs (10, 25, and 50 mg/Kg/7 days) (Liu et al., 2013). Regarding *in vitro* studies, the exposure of human (SK-N-SH) and mouse (Neuro-2a) neuroblastoma cells to silica NPs (SiNPs) (10 µg/mL/24 h) raised the intracellular content of Aβ in both cell lines, which was associated with increased APP and reduced NEP protein levels. These effects may be mediated by ROS production, since SiNPs increased the production of intracellular ROS (Yang et al., 2014). Likewise, treatment of Neuro-2a cells to silver NPs (AgNPs, 12.5 µg/mL/24 h) showed the deposition of Aβ plaques and an increased expression of *APP*, while NEP and LPR1 (or LDLR) expression and protein levels were reduced, suggesting that AgNPs can induce AD by altering the amyloidogenic pathway: Aβ synthesis, degradation or clearance (Huang et al., 2015). Interestingly, authors also reported an increased expression of genes involved in the inflammatory response such as *IL-1*, C-X-C motif chemokine 13 (*CXCL13*), macrophage receptor with collagenous structure (*MARCO*), and glutathione synthetase (*GSS*) (Huang et al., 2015).

#### Tau Hyperphosphorylation by Environmental Factors

Several environmental factors have shown to mediate AD development through alterations on Tau phosphorylation and/or aggregation (Figure 1).

##### Metals

*In vivo* and *in vitro* studies have suggested the potential of Hg to induce P-Tau. Fujimura et al. (2009) reported an increased neuronal death and more migrating astrocytes in cerebral cortex of male mice exposed to MeHg (30 ppm, drinking water), as well as increased levels of P-Tau mediated by c-jun N-terminal kinase (JNK; Fujimura et al., 2009). An *in vitro* study showed that inorganic Hg (50 µg/dL/30 min) increased P-Tau in SH-SY5Y cells by a ROS-dependent mechanism, which was reverted by the co-treatment with the antioxidant melatonin (Olivieri et al., 2000). Another study demonstrated that Hg ions coordinate with Cys291 of the second repeated (R2) of the MT-binding domain of Tau increasing the heparin-induced aggregation, and a conformational change in Tau demonstrated by circular dicroism (CD) analysis (Yang et al., 2010). On the other hand, Cd appears to play a role in Tau hypothesis since it promotes the aggregation of this protein. It was shown that Cd (II) accelerates heparin-induced aggregation of the third repeated (R3) of Tau. The binding of Cd (II) to the dimeric R3 produces changes on its conformation demonstrated by CD (Jiang et al., 2007). Subchronic As administration to rats (NaAsO_2_ at 3 and 10 mg/Kg/day/4–12 weeks, intragastric) induced P-Tau, suggesting that As-destabilization and disruption of the cytoskeletal framework may lead to axonal degeneration (Vahidnia et al., 2008). Regarding Pb, it was reported that infantile Pb exposure in cynomolgus monkeys elevated mRNA and protein levels of Tau as well as its transcriptional regulators (Sp1 and Sp3) in aged primates (23 years old). An increase in P-Tau phosphorylation and mRNA and protein levels of cyclin dependent kinase 5 (cdk5, a kinase that phosphorylates Tau) were also observed (Bihaqi and Zawia, 2013). Other studies also reported that maternal (Li et al., 2010a) and early postnatal exposures (Liu et al., 2014) to Pb produced significant increased P-Tau levels and cognitive impairment in mice. Finally, chronic Al exposure caused Tau aggregation, and it was suggested that Al is bound to P-Tau in the Al-NFTs lesions (Singer et al., 1997; Shin et al., 2003). Also, a study showed that Al is able to confer resistance to the degradation of PHFs both *in vivo* and *in vitro* (Shin et al., 1994), and it can inhibit the activity of the protein phosphatase 2 (PP2), which is involved in P-Tau de-phosphorylation (Yamamoto et al., 1990).

##### Pesticides

There is some evidence suggesting that pesticide exposure can disrupt Tau function. A recent study showed that the administration of the insecticides deltamethrin (pyethroid) and carbofuran (carbamate) to rats (daily administration by gavage/28 days) produced neuronal death in the cortex and hippocampus and a dysfunction in the spatial memory and learning. These alterations were attributed to a reduced expression of synaptic proteins involved in the memory consolidation. Additionally, P-Tau and activation of p-GSK3β (a major kinase that phosphorylates Tau) were observed (Chen et al., 2012b). Similarly, Wills et al. (2012) showed P-Tau in the striatum, through the activation of p-GSK3β, as well as hyperacetylation of α-tubulin in mice treated with PQ (10 mg/Kg, i.p., twice weekly/6 weeks), suggesting a cytoskeleton remodeling (Wills et al., 2012).

##### Nanoparticles

The effect of NPs on Tau phosphorylation has not been extensively studied. Silica NPs (siNPs) used in medicine are also able to increase P-Tau at Ser262 and Ser396, two phosphorylation sites characteristic of AD. It was demonstrated that this effect was dependent on the activation of the kinase GSK3β in human SK-N-SH and mouse Neuro-2a cells by a mechanism probably mediated by oxidative stress, since ROS were increased in cells exposed to these NPs (Yang et al., 2014).

### Parkinson’s Disease

Parkinson Disease is a chronic and progressive neurological disorder characterized by the selective loss of dopaminergic neurons of the substantia nigra pars compacta (SNpc). The cardinal features of the syndrome are related to motor dysfunction including tremor at rest, rigidity, akinesia (or bradykinesia), and postural instability. The motor symptoms appear when at least 60% of dopaminergic neurons are lost and 80–85% of dopamine content in the striatum is depleted (Jankovic, 2008; Wirdefeldt et al., 2011). Additional to the neuronal loss, the main neuropathological hallmark of PD is the presence of Lewy bodies (LB) in the surviving neurons, which are eosinophilic cytoplasmic inclusions containing aggregates of protein such as α-synuclein (α-syn) (Gibb and Lees, 1988; Spillantini et al., 1998). PD is the second most common neurodegenerative disorder after AD. Due to the lack of specific/differential diagnostic biomarkers, the diagnosis of PD is based on clinical criteria of specific cardinal motor signs of the disease and on the response to levodopa. PD diagnosis is confirmed by the depletion of brain stem pigmented neurons and the presence of LB at necropsy, this is the reason of the misclassification of PD cases (about 10–15%) (Schrag et al., 2002; Jankovic, 2008). There is no cure for PD, and the existing therapies only provide brief relief of motor symptoms through improving the dopamine deficit or by surgical methods. This highlights the need of research on early specific/differential biomarkers to have more accurate diagnosis of neurodegenerative disorders, as well as biomarkers for the identification of populations at risk to implement neuroprotective therapies (Jankovic, 2008).

As in the case of AD, circulating miRNAs are being studied as differential biomarkers for PD. Some reviews have recently addressed this topic, showing the association of specific miRNAs for some genes involved in PD, such as *SNCA* and *LRRK2* (encoding for leucine–rich repeat kinase 2) with PD development (Goodall et al., 2013; Maciotta et al., 2013). Some studies have reported differentially expressed miRNAs in serum of PD patients not observed in control subjects or in other diseases. For example, Vallelunga et al. (2014) reported two differentially expressed miRNAs (miR-30c and miR-148b) in Italian PD patients (*n* = 25 vs. 25 healthy controls) (Vallelunga et al., 2014), and another study found that serum levels of miR-29c, miR-29a, and miR-19b were down-regulated in PD patients (*n* = 65 vs. 65 healthy controls) from Barcelona, Spain (Botta-Orfila et al., 2014). Also, a reduced expression of miR-34b and miR-34c in several brain areas including the substantia nigra of PD patients (*n* = 11 vs. 6 healthy controls) was detected; interestingly the misregulation of miR-34b/c was observed in patients in pre-motor stages of the disease. Additionally, these miRNAs were deregulated in differentiated SH-SY5Y dopaminergic neuronal cells, which was associated with altered mitochondrial function, oxidative stress and ATP depletion, as well as decreased protein levels of DJ1 (a mitochondrial peroxidase) and Parkin (an E3 ubiquitin ligase) that are associated with the familial form of PD (Miñones-Moyano et al., 2011).

Although the research on PD has rapidly advanced, the molecular mechanisms involved are still unclear and its etiology is complex. Several molecular mechanisms of neuronal death in PD pathogenesis have been described including mitochondrial dysfunction, impairment of protein quality pathways, oxidative/nitrative stress, microglia activation and inflammation. These mechanisms converge and are consistent with a major role of oxidative stress in PD, which damage organelles and proteins leading to increased protein aggregates (e.g., α-syn), that in turn overwhelms the degradation systems leading to a self-perpetuating cycle and further oxidative stress (Wirdefeldt et al., 2011; Goldman, 2014). The evidence in postmortem PD brains supports these mechanisms, as well as a decreased in reduced GSH levels, α-syn aggregation, proteasome impairment and autophagy dysfunction (review in Navarro-Yepes et al., 2014).

**Figure 2:**
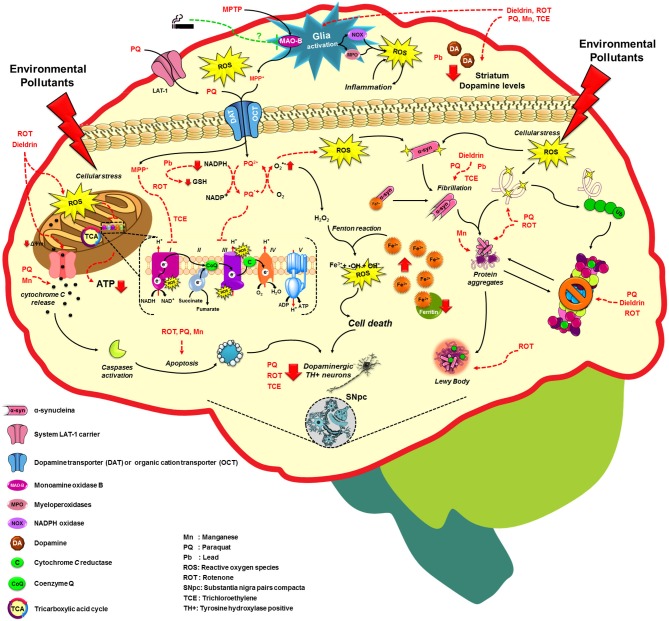
**Molecular mechanisms altered by environmental factors related to increased Parkinson’s disease risk**. Exposure to environmental toxicants mainly pesticides, metals and solvents may lead to the selective loss of dopaminergic neurons on the substantia nigra pars compacta (SNpc) through the dysregulation/alteration of the molecular mechanisms implicated on PD development such as mitochondrial dysfunction, impairment of protein quality pathways, microglia activation and inflammation, which converge in the production of oxidative stress as the main factor in PD. For more detail see the text.

A fraction of PD occurrence has a clear familial inheritance and it is related to mutations in at least 6 genes that have been associated with PD onset. The identification of genes such as *SNCA* or* PARK1* encoding for α-syn (maybe involved in the regulation of dopamine release and transport), *LRRK2* or* PARK8* encoding for LRRK2 (or Dardarin), *PARK7* encoding for DJ1, *PARK6* or* PINK1* encoding for PTEN-induced putative kinase 1 (PINK1, a mitochondrial kinase), and *PARK2* encoding for Parkin have provided clues about the molecular mechanisms involved in its pathogenesis (Corti et al., 2011; Cookson, 2012). However, 90% of PD cases are sporadic and cannot be attributed only to genetic factors, which suggests that PD have a multifactorial etiology (Goldman, 2014). In addition to the aging, which is the main risk factor for PD (Tanner and Goldman, 1996), epidemiological evidence suggests that the exposure to environmental toxicants, mainly pesticides, metals and solvents could increase the risk of developing PD, and factors such as tobacco consumption can protect against PD development (Figure 2; Hatcher et al., 2008; Gao and Hong, 2011).

#### Metals

It has been proposed that chronic exposure to heavy metals such as iron, Pb and Mn and their combinations can be associated with an increased risk of developing PD, since they accumulate in the substantia nigra and generate oxidative stress. However, epidemiological evidence is controversial (Lai et al., 2002). The epidemiological evidence of Pb association with PD is more consistent because the accumulative lifetime exposure can be estimated through Pb concentration in bone that has a half–life of years to decades. Initially, Kuhn et al. (1998) reported that 7 out of 9 postal workers exposed to lead-sulfate batteries for up to 30 years developed parkinsonian symptoms, suggesting that Pb intoxication may play a role in the occurrence of these symptoms (Kuhn et al., 1998). Coon et al. (2006) evaluated this association in 121 PD patients vs. 414 controls and found that chronic Pb exposure (evaluated by Pb concentrations in tibial and calcaneal bones) increased 2–fold the risk of PD (OR = 2.27, 95% CI, 1.13–4.55) for individuals in the highest quartile of lifetime Pb exposure relative to those in the lowest quartile (Coon et al., 2006). In the same way, it was reported that the cumulative exposure to Pb increases the risk of PD (OR = 3.21, 95% CI, 1.17–8.83) in 330 PD patients (vs. 308 controls) recruited from 4 clinics for movement disorders in Boston, MA area (Weisskopf et al., 2010b), and the exposure to Pb for more than 20 years showed a stronger association with PD risk in a health system population-based case-control study (144 cases vs. 464 controls) from the metropolitan Detroit area (Gorell et al., 2004). At the molecular level, Pb exposure significantly decreases the dopamine release and the dopamine D1 receptor sensitivity post-synaptically in microdialysate samples from rats subchronically exposed to Pb (50 ppm/90 days) (Kala and Jadhav, 1995), and in rats treated with 250 ppm of Pb for 3–6 weeks through drinking water (Tavakoli-Nezhad and Pitts, 2005). Furthermore, it increases the lipid peroxidation and reduces the antioxidant cell capacity (Sandhir et al., 1994), and causes fibrillation and aggregation of α-syn (Yamin et al., 2003), which induces hippocampal injury and decreases the ability of learning and memory in rats exposed to 0–300 ppm of Pb (Yamin et al., 2003; Zhang et al., 2012).

Manganese is an essential element with important physiological functions for cellular homeostasis. The epidemiologic evidence does not provide sufficient support for an association between Mn exposure and PD risk (Wirdefeldt et al., 2011; Mortimer et al., 2012). Only one case–control study (144 cases vs. 464 controls) in a population from the metropolitan Detroit area reported an increase of PD risk when the exposure to Mn was over 20 years (OR = 10.63, 95% CI, 1.07–105.99) (Gorell et al., 2004). However, occupational or environmental exposures to Mn have been associated with a neurological syndrome that include cognitive deficits, neuropsychological abnormalities and Parkinsonism (Guilarte, 2013). Mn was related to PD since 1837, when it was noted that high Mn exposures caused a severe and debilitating disorder known as “manganism” or manganese–induced Parkinsonism, which consists on an extra pyramidal syndrome that resembles the dystonic movements associated with parkinsonian symptoms (Couper, 1837; Jankovic, 2005), but it is clinically distinct from PD since patients do not respond to dopamine replacement therapies (Cook et al., 1974; Huang et al., 1993; Lu et al., 1994). Several cases of Mn–induced Parkinsonism have been reported in individuals whose professions involve prolonged contact with high atmospheric levels of Mn such as welders, miners and smelters (Rodier, 1955; Wang et al., 1989; Lee, 2000). Several investigations have shown that sustained exposure to low-concentrations (below the current US standard of 5.0 mg/m^3^) is consistent with early manganism, suggesting that Mn is a neurotoxic chemical (Park, 2013). Patients with manganism and primates experimentally intoxicated with Mn consistently show damage to the globus pallidus, which is in contrast with PD where there is a preferential degeneration of dopamine neurons in the SNpc and preservation of the pallidum (Perl and Olanow, 2007). Likewise, it was observed microglia activation in the substantia nigra pars reticulate (SNpr) and SNpc in *Cynomolgus macaques* exposed to Mn (5–6.7 mg/Kg/week/10 months) (Verina et al., 2011). *In vitro*, it has been observed that Mn treatment (50–300 µM MnCl_2_/ 3–48 h) induces cytochrome *C* release and activation of caspases 9 and 3, as well as protein aggregation in N27 dopaminergic neuronal cells that stably express α-syn (Harischandra et al., 2015).

Iron is an essential element transported into the brain through the transferrin receptor and divalent metal transporter 1 (DMT1; Zheng and Monnot, 2012). It has been evaluated in relation to the risk of PD in few epidemiological studies without convincing evidence (Rybicki et al., 1993; Logroscino et al., 2008; Miyake et al., 2011; Abbott et al., 2012). However, iron and its deregulated homeostasis have been proposed to have a role in the pathogenesis of PD because of its pro-oxidants characteristics that may lead to ROS generation via Fenton and Haber–Weiss reactions (Stohs and Bagchi, 1995; Sian-Hulsmann et al., 2011). The substantia nigra has the highest levels of iron in the human brain, probably due to the presence of neuromelanin in pigmented SNpc dopaminergic neurons that have an impressive capacity of chelating metals, iron in particular; however, this may be a dual-edged sword that may increase their vulnerability since iron may react with ROS produced from dopamine metabolism and promote the further generation of highly toxic radicals (Zecca et al., 2002, 2004). Alterations in iron distribution have been observed in the substantia nigra of PD postmortem brains (Dexter et al., 1987, 1991; Hirsch et al., 1991). On the other hand, it was observed in postmortem samples that although the total iron concentration in the whole substantia nigra was not significantly different between parkinsonian and control samples, there was an increase in the free-iron concentration and a decrease in iron–binding ferritin levels, ferritin sequestrates the excess of iron under physiological conditions (Wypijewska et al., 2010). Likewise, it was reported that free-iron induces fibrillation and aggregation of α-syn in a dose- and time-dependent way in SK-N-SH cells incubated with ferric iron (1–10 mmol/L/24–48 h) (Li et al., 2010b). Mice administered with iron (120 µg/g of carbonyl iron, oral gavage) at a dose equivalent to that found in iron-fortified human infant formula (12 mg/L of iron) from days 10 to 17 post-partum (an equivalent period to the first human year of life) showed a progressive midbrain neurodegeneration and enhanced vulnerability to toxic injury at 12 and 24 months of age (Kaur et al., 2007).

#### Pesticides

The hypothesis that pesticide exposures may be related to PD development was prompted by the discovery that intravenous injection of 1-methy l-4pheny l-1, 2, 3, 6-tetrahydropyridine (MPTP), a byproduct of the synthesis of heroin, developed a Parkinson syndrome clinically indistinguishable from PD (Langston et al., 1983); subsequent findings showed that MPTP selectively damaged dopaminergic neurons in the substantia nigra (Langston and Ballard, 1983; Langston et al., 1984). Since then, environmental factors with similar toxicological profiles have received attention as potential risk factors for PD.

A meta-analysis conducted in 2000 evaluated the association between pesticide exposures and PD in 19 case–control studies published between 1989 and 1999. Authors showed that most studies found an elevated risk of PD with the exposure to pesticides, the calculated combined OR was 1.94 (95% CI, 1.49–2.53); similar ORs were observed in studies conducted in United States, Asia, Europa and Canada. Additionally, it was observed that the risk of PD increased with longer exposure times, with an OR of 5.81 (95% CI, 1.99–16.97) for ≥10 years of exposure; however, specific types of pesticides were not identified (Priyadarshi et al., 2000). Subsequently, Brown et al. (2006) reviewed 31 case–control studies published until 2003, and found that about half of them reported significant associations between pesticide exposure and PD risk with ORs from 1.6–7. Interestingly, in most studies, authors observed a positive association between the exposure to herbicides and insecticides and PD risk, but not with the exposure to fungicides alone (Brown et al., 2006).

In line with this, a recent review by Freire and Koifman (2012) analyzed the epidemiological evidence published between 2000 and 2011, including ecological, cross–sectional, prospective and case–control studies. They found that 7 out of the 8 prospective (cohort) studies provided evidence of an association between pesticide exposure and PD, reporting risk estimates of 2-fold or higher. Among 23 case–control studies, 13 studies reported a significant increased risk of PD for the professional use of pesticides in comparison with unexposed controls, with ORs ranging from 1.1 to 1.4, which is in agreement with the review of Priyadarshi in the 1990’s (Freire and Koifman, 2012). Furthermore, van der Mark et al. (2012) performed a systematic review and calculated the summary risk ratio (sRR) from 39 case–control studies, 4 cohort studies and 3 cross–sectional studies. When a job–exposure matrix was constructed, a higher sRR (2.5, 95% CI, 1.5–4.1) was observed compared with self–reported exposure evaluation (1.5, 95% CI, 1.3–1.8). This meta–analysis found a positive association between PD and insecticides (sRR = 1.50, 95% CI, 1.07–2.11), and herbicides (sRR = 1.40, 95% C, 1.08–1.81), but not with fungicides (sRR = 0.99, 95% CI, 0.71–1.40) (van der Mark et al., 2012), in agreement with Brown et al. (2006) and Freire and Koifman (2012). Other factors related to pesticide exposure such as well–water consumption, farming, and rural living have been associated with an increased PD risk. The meta–analysis of Priyadarshi et al. (2001) found a combined OR of 1.56 (95% CI, 1.18–2.07) for rural living, 1.42 (95% CI, 1.05–1.91) for farming and 1.26 (95% CI, 0.97–1.64) for well–water consumption. However, whether of these factors are independent risk factors or correlated with pesticide exposure could not be determined (Priyadarshi et al., 2001).

In support to epidemiological evidence, increased levels of some pesticides have been quantified in postmortem brains from PD patients. High concentrations of some OCl pesticides have been observed in PD cases compared with controls, including dieldrin, lindane, and *p-p*-DDE (Fleming et al., 1994; Corrigan et al., 1998, 2000). In the same way, 2 epidemiologic studies reported a significant association (OR ranging from 1.3 to 1.8) between dieldrin use and PD in farmers participants in the Agricultural Health Study (AHS; Kamel et al., 2007; Tanner et al., 2011). Another nested case–control study within the Finnish Mobile Clinic Health Examination Survey in Finland, with serum samples collected during 1968–1972, observed that increasing serum concentrations of dieldrin were associated with an increased PD risk (OR = 1.95, 95% CI, 1.26–3.02) in 68 cases vs. 183 controls restricted to never smokers, while no other OCl pesticide showed an association (Weisskopf et al., 2010a).

The epidemiologic evidence that dieldrin exposure may be associated with PD is supported by toxicological data at molecular level. Dieldrin may cross the blood–brain barrier and remains in lipid-rich tissues such as the brain (Kanthasamy et al., 2005), and it has been shown that it is selectively toxic to dopaminergic neurons and could induce several of the pathologic mechanisms of PD including the depletion of brain dopamine levels, increased ROS in nigral dopaminergic neurons, inhibition of mitochondrial oxidative phosphorylation that lead to a reduction of cellular ATP production, alteration of the mitochondrial membrane potential and cytochrome *C* release in animal models such as rats and mice chronically exposed to dieldrin (0.3–3 mg/Kg/day in the diet) (Bergen, 1971; Wagner and Greene, 1978; Purkerson-Parker et al., 2001; Hatcher et al., 2007), and in primary mesencephalic cultures or dopaminergic cell lines (0.01–300 µM) (Sanchez-Ramos et al., 1998; Kitazawa et al., 2001, 2003; Kanthasamy et al., 2005). Aggregation of α–syn, ubiquitin–proteasome impairment function (Uversky et al., 2001; Sun et al., 2005) and microglia activation (Mao and Liu, 2008) have also been observed.

Paraquat is a quaternary nitrogen herbicide used worldwide. Due to its structural similarity to MPP (the active metabolite of MPTP), it was thought to be toxic to dopaminergic neurons and thus might be related to PD. The possible association between PQ and PD received attention from the study of Liou et al. (1997) performed in PD patients (120 patients and 240 controls) in Taiwan, in which the pesticide use was associated with an increased risk of developing PD, being higher for those individuals who reported using PQ (Liou et al., 1997). Likewise, Tanner et al. (2011) reported a significant association between PD and the use of oxidative pesticides, including PQ (OR = 2.5, 95% CI, 1.4–4.7) in professional pesticide applicators (110 cases and 358 controls) (Tanner et al., 2011). Similarly, other epidemiologic studies have associated the exposure to PQ with PD (Hertzman et al., 1990; Ascherio et al., 2006; Kamel et al., 2007; Wang et al., 2011).

Paraquat is taken up into dopaminergic terminals by the dopamine transport and organic cation transporter 3 (Rappold et al., 2011), and causes cellular toxicity by oxidative stress through the cellular redox cycling generating superoxide radical by the oxidation of NADPH, which in turn impairs the restauration of GSH levels and thus the activity of several antioxidant systems (Berry et al., 2010; Franco et al., 2010). It has been observed that repeated administrations of PQ to adult mice and rats (5–10 mg/Kg/ week/at least 3 weeks, i.p.) increase ROS levels in the striatal homogenate, induce a dose-dependent decrease in dopaminergic neurons from the substantia nigra, a decline in striatal dopamine nerve terminal density, and a neurobehavioral syndrome characterized by reduced ambulatory activity (Brooks et al., 1999; McCormack et al., 2002; Kuter et al., 2010). PQ also reproduces other biochemical and neuropathological characteristics of human Parkinsonism such as microglia activation (Wu et al., 2005; Purisai et al., 2007), α-syn up-regulation and fibrillation (Uversky et al., 2001; Manning-Bog et al., 2002), increases lipid peroxidation (increase of 4-hydroxynonenals) (McCormack et al., 2005), alters parkin solubility promoting its intracellular aggregation (Wang et al., 2005), induces a proteasome dysfunction in SH-SY5Y cells (Ding and Keller, 2001; Yang and Tiffany-Castiglioni, 2007), as well as in homogenates from postmortem PD brains (McNaught and Jenner, 2001; McNaught et al., 2002), impairs mitochondrial function at the level of complex III to generate ROS (Castello et al., 2007; Drechsel and Patel, 2009), promotes cytochrome *C* release (González-Polo et al., 2004; Fei et al., 2008), induces GSH depletion (Schmuck et al., 2002; Kang et al., 2009), and causes cell injury leading to apoptotic cell death (Berry et al., 2010; Franco et al., 2010). PQ has been used as a toxicological model for PD that has permitted getting important information about the mechanisms involved in the neurodegeneration associated with PD (Gao and Hong, 2011).

Rotenone, an OP insecticide has also been associated with an increased risk of PD. Two epidemiological studies found an association between rotenone exposure and PD risk, reporting an increased risk of 10–fold (OR = 10.0, 95% CI, 2.9–34.3) in East Texas farmers (Dhillon et al., 2008), and 2.5-fold (OR = 2.5, 95% CI, 1.3–4.7) in PD cases (*n* = 110) compared with controls (*n* = 358) from professional pesticide applicators participants in the AHS (Tanner et al., 2011). Rotenone can freely cross the blood–brain barrier and is a well-established mitochondrial toxin that specifically inhibits the complex I (NADH–dehydrogenase) of the electron transport chain leading to ATP depletion, energy failure and mitochondrial ROS production, which in turn induces cytochrome *C* release and apoptotic cell death (Clayton et al., 2005; Radad et al., 2006; Sherer et al., 2007). It has been shown that, like MPTP, rotenone treatment in animal models (1.5–3 mg/Kg/day/up to 3 weeks) reproduces features of PD such as bradykinesia, postural instability and/or rigidity, reduces the tyrosine hydroxylase-positive neurons in the substance nigra, induces a loss of striatal dopamine, and the accumulation of α-syn and poly-ubiquitin positive aggregates in remaining dopaminergic neurons (Betarbet et al., 2000; Sherer et al., 2003; Cannon et al., 2009). Likewise, Betarbet et al. (2006) observed that chronic administration of 3.0 mg/Kg/day of rotenone for up to 5 weeks to male rats caused the oxidation of DJ-1, accumulation of α-syn, and proteasomal impairment (Betarbet et al., 2006). These effects were also observed in neuroblastoma SK-N-MC cells treated with rotenone (5 nM/4 weeks), as well as a loss of GSH, oxidative DNA and protein damage and caspase-dependent death (Sherer et al., 2002; Betarbet et al., 2006). Rotenone has also the capacity to activate microglia (Sherer et al., 2003); Gao et al. (2002) demonstrated that the addition of microglia to primary neuron-enriched cultures (neuron/glia cultures) markedly increased the dopaminergic neurodegeneration induced by rotenone (1 nM/8 days), and this neurotoxicity was attenuated by the inhibition of NADPH oxidase or scavenging the superoxide radical that is liberated from the microglia (Gao et al., 2002). Since rotenone recapitulates several mechanisms of PD pathogenesis, this pesticide is currently used as a toxicological model to study the underlying mechanisms on the PD development.

Despite the widespread use of OP insecticides such as malathion, methyl parathion, chlorpyriphos and diazinon, not many studies have evaluated the association between specific OP and PD risk. Dhillon et al. (2008) found a 2–fold increase (OR = 2.0, 95% CI, 1.02–3.8) in the risk of PD in Texan agricultural workers exposed to chlorpyriphos (cases = 100, controls = 84) (Dhillon et al., 2008). An increased risk of PD was also observed in rural residents from California possibly exposed to high levels of chlorpyriphos (OR = 1.87, 95% CI, 1.05–3.31) and diazinon (OR = 1.75, 95% CI, 1.12–2.76) through the consumption of contaminated well–water (Gatto et al., 2009). One study conducted in a population from the Group Health Cooperative (GHC) in Western Washington State occupationally exposed to methyl parathion found a high risk of PD (OR = 8.08, 95% CI, 0.92–70.85), although the association was not statistically significant (Firestone et al., 2005). This is particularly relevant, because parkinsonian effects have been reported in cases of patients intoxicated with OP (Bhatt et al., 1999).

#### Solvents

Solvents are widespread used due to their commercial applications, including metal degreasing, dry cleaning, and as ingredients of paint thinners and detergents. Some solvents are lipophilic and thus easily absorbed by the central and peripheral nervous system tissues (Lock et al., 2013). There are isolated cases of acute Parkinsonism associated with large solvent exposures such as in workers exposed to *n*-hexane (Pezzoli et al., 1989), and toluene (Papageorgiou et al., 2009), among others. There is no consistent evidence of the association of solvent exposure and PD (Wirdefeldt et al., 2011). One case-control study based on a questionnaire reported an increased risk of PD by the exposure to organic solvents (OR = 2.78, 95% CI, 1.23–6.26) in 86 PD patients and 86 controls from the Emilia-Romagna region in Italy (Smargiassi et al., 1998). Another case–control study reported an increased risk of PD when the exposure to solvents was above 20 years (OR = 3.59, 95% CI, 1.26–19.26) in 182 cases (vs. 422 controls) identified through death certificates of the Rolls-Royce PLC national pension fund archive from employees of five manufacturing locations in United Kingdom who had any mention of PD (McDonnell et al., 2003).

Trichloroethylene (TCE) is one of the specific solvents that has been investigated in detail (Goldman, 2014). Some clinical case reports have reported the onset of PD in workers exposed to TCE through chronic inhalation and dermal exposure by handling TCE, suggesting a potential link between the exposure to TCE and PD (Kochen et al., 2003; Gash et al., 2008). More recently, an epidemiologic study in 99 twin pairs discordant for PD showed that the exposure to TCE was associated with a 6–fold increased risk of PD (OR = 6.1, 95% CI, 1.2–33) (Goldman, 2014). In animal models, TCE may recapitulate several key pathological features of PD. The systemic exposure of adult rats to TCE (1000 mg/Kg/day/5 days a week/2 and 6 weeks, oral gavage) inhibits mitochondrial complex I enzyme activity, increases oxidative stress markers, activates the microglia, induces nigral α-syn accumulation and a significant loss of dopaminergic neurons on the SNpc in a dose-dependent manner, as well as defects in the rotarod behavior test (Liu et al., 2010). In a similar way, the administration of *n-*hexane and its metabolite 2, 5-hexanedione (400 mg/Kg/day/5 days a week/6 weeks, i.p.) to mice caused that both chemicals reduced the striatal dopamine concentration by 38 and 33%, respectively, but neuronal cell loss was not confirmed (Pezzoli et al., 1990). On the other hand, there is no evidence that acute or subchronic exposure to toluene promotes the degeneration of the nigrostriatal dopamine system (Lock et al., 2013).

#### Nanoparticles and PD

Nanoparticles are an important alternative in the development of treatment strategies for neurodegenerative diseases due to their small particle size, large surface and high drug loading efficiency, which allow them to cross the blood-brain barrier and efficiently release specific drugs (Li et al., 2014b; Leyva-Gómez et al., 2015). However, their small size allows them to penetrate the cell and organelles, disrupting their normal function (Buzea et al., 2007).

Although some NPs are being used in therapies for PD, no epidemiological studies are available associating them with PD risk. However, there is evidence suggesting that they could contribute to alter the molecular mechanisms involved in the pathogenesis of PD. Thus, it was reported that intranasal instillation of SiO_2_-NPs (20 µg/day/1–7 days) to rats resulted in their presence in the striatum, the induction of oxidative damage, an inflammatory response, and depleted dopamine concentration and tyrosine hydroxylase levels, suggesting that these NPs have a negative impact on striatal dopaminergic neurons (Wu et al., 2011). Another report in adult zebrafish exposed to SiO_2_-NPs (300 and 1000 µg/mL; 15 and 50-nm of size) showed alterations in neurobehavioral parameters (general, cognitive behavior and locomotive activity), with the most significant effects observed with the smallest NPs, similar to those observed in neurodegenerative diseases (Li et al., 2014b). *In vitro* studies also support the potential contribution of NPs in PD development. The exposure of dopaminergic neurons (PC12 cells) to SiO_2_-NPs (25–200 µg/mL/24 h) triggered an oxidative stress, disturbed the cell cycle, induced apoptosis, and activated the p53-mediated signaling pathway (Wu et al., 2011); while the exposure of these cells to TiO_2_-NPs (50, 100 and 200 mg/mL/24 h) induced a dose–dependent increase in the expression and aggregation of α-syn, as well as a reduction of the expressions of *Parkin* (E3 ligase), and the ubiquitin C-terminal hydrolase (*UCH-L1*), these events were associated with increased oxidative stress (Wu and Xie, 2014). Also, the exposure PC12 cells to iron oxide (Fe_2_O_3_-NPs; 0.15–15 mM) decreased the neurite growth in response to the nerve growth factor (NGF) (Pisanic et al., 2007). Likewise, citrate-capped gold nanoparticles (Au-NPs; 0.3–32 nM, 10–22 nm) produced a dose-dependent aggregation of purified α-syn, being strongest for the smallest NPs (Alvarez et al., 2013). In contrast, the administration of Neurotensin (NTS)-polyplex NPs (8.5 nmol/Kg, i.v), a nanocarrier gene with a potential for nanomedicine-based applications for PD treatment, to BALB/c mice does not produce systemic inflammatory (up to 24 h after treatment) nor hepatic cytotoxicity (at 24 and 96 h after treatment), supporting the safety of these NTS-polyplex NPs as a potential therapeutic approach (Hernandez et al., 2014).

### Early Exposure to Environmental Factors and AD or PD Development: Epigenetic Evidence

Epigenetic DNA modifications include DNA methylation, histone post-translational modifications (mainly acetylation) and miRNAs (Holliday, 2006). DNA methylation is one of the most studied epigenetic modifications that influence the gene expression. It involves the addition of methyl groups to cytosine bases located at cytosine–phosphate–guanine (CpG) sites by the action of DNA methyltransferases (DNMTs). Alterations in DNA methylation on the promoter regions of genes regulate the gene expression of important processes such as embryonic development, cellular differentiation and aging (Bird, 2002). Increasing evidence suggests that epigenetic changes in the developing embryo that may play important roles in the susceptibility to diseases in later life (imprinted disease phenotypes) result from maternal exposures to environmental stimuli at critical periods of development. This suggests that a short exposure to chemicals could be memorized through epigenetic mechanisms long after the chemical trigger has gone (Jang and Serra, 2014), and recent studies have suggested that an epigenetic component could be involved in neurodegenerative diseases related to environmental factors (Marques et al., 2011).

The latent brain expression of genes observed in animals developmentally exposed to an environmental contaminant may be mediated through epigenetic pathways that are regulated via the DNA methylation. While the conditions leading to early life hypo- or hyper-methylation of specific genes are not known, both can induce oxidative DNA damage; for instance the hypo-methylation of *APP* gene increases its expression driving the overproduction of APP and Aβ levels, which in turn facilitate the ROS production damaging the DNA, and producing neuronal loss. While the hyper-methylation affects the gene transcription and DNA repair pathways. Therefore, both changes in DNA methylation can impact gene expression and imprint susceptibility to oxidative DNA damage in the aged brain (Zawia et al., 2009). Thus, it is suggested that Pb interferes with the DNA methylating capacity, thus altering the expression of AD-related genes. The study performed in aged monkeys developmentally exposed to Pb revealed a reduced activity of brain Dnmt, and the exposure of mouse primary cells from the cerebral cortex to Pb (0.1 µM) resulted in a similar effect on Dnmt1 activity a week after 24 h-treatment (Wu et al., 2008). Also, Bihaqi and Zawia (2012) showed a significant latent increase in AD biomarkers an a reduction in the protein and mRNA levels of DNA methylating enzymes Dnmt1 and Dnmt3a, and methyl CpG binding protein 2 (MeCP2) in differentiated SH-SY5Y cells treated with Pb (5–100 µM/48 h) and analyzed 6 days later (Bihaqi and Zawia, 2012). Aberrant CpG methylation in *APP*, *Tau* and *GSK3*β genes was reported in post-mortem brains (Iwata et al., 2014). In addition, it suggested that reduced levels of CpG methylation in the promoter of *APP* could be mediated by the oxidation of guanine (8-oxdG) (Zawia et al., 2009); this is because the oxidation of guanine in CpG dinucleotides inhibits adjacent cytosine methylation (Weitzman et al., 1994). On the other hand, Cd, another metal involved in AD pathology, reduces the enzymatic activity of Dnmt in rat liver cell cultures (Poirier and Vlasova, 2002), but this effect has not been evaluated in cerebral cells. While a study showed that subchronic As exposure (3 and 36 ppm/from gestation until 4 months of age) altered the methylation of genes involved in neuronal plasticity, including reelin (RELN) and protein phosphatase 1 (PP1), which was associated with memory deficits (Martínez et al., 2011). Regarding other compounds, the perinatal exposure to permethrin (34 mg/Kg/daily, by gavage from postnatal day 6–21) to mice showed altered brain functions including biomarkers of maintenance of dopaminergic neurons, and impairment of spatial memory at 6 months of age (Nasuti et al., 2013).

The relation between epigenetic modifications and PD has been less studied; however, a potential role of DNA methylation in the promoter of α-syn encoding gene (*SNCA*) in the neuropathogenesis of PD has been suggested, considering that α-syn is a fundamental component of LB, the main hallmark of PD (Lu et al., 2013). A DNA hypomethylation of *SNCA* was reported in the substantia nigra of sporadic PD patients, suggesting that it might contribute to the dysregulation of *SNCA* expression in PD (Jowaed et al., 2010; Matsumoto et al., 2010). In addition, increased *SNCA* mRNA levels were observed in SNpc of PD (Chiba-Falek et al., 2006), and reduced levels of Dnmt1 have been observed in postmortem brains from PD and dementia with LB (DLB) patients, as well as in brains of α-syn transgenic mice; authors suggest that this effect could be a novel mechanism of epigenetic dysregulation in LB-related diseases such as PD (Desplats et al., 2011). Finally, a lesser degree of methyation of the *TNF*α promoter, a key inflammatory cytokine associated with dopaminergic cell death was observed in the SNpc from PD patients, predisposing to an increase neuronal vulnerability to inflammatory reactions (Mogi et al., 1996; Pieper et al., 2008).

Environmental factors associated with an increased risk of PD such as pesticides can alter the expression of genes by epigenetic mechanisms (Kwok, 2010). It was reported that pre-treatment with 5-aza-2’deoxycytidine (5’-aza-dC, a DNMT inhibitor) exacerbated the dopaminergic neuron damage induced by PQ, MPP^+^, 6-hydroxydopamine (6-OHDA) and rotenone treatment, and induced oxidative stress, the transcriptional up-regulation of α-syn, and demethylation of the α-syn promoter (Wang et al., 2013). Likewise, the folate deficiency sensitizes mice to MPTP-induced PD-like pathology and motor dysfunction (Duan et al., 2002); it is well known that folate deficiency alters the development of human nervous system (Greenblatt et al., 1994).

On the other hand, it was reported that the exposure to environmental neurotoxicants associated with PD during early life or pregnancy can determine the progressive damage of the substantia nigra years before the onset of clinical parkinsonism, as well as to increase the vulnerability to effects of a second environmental factor (two–hit model) (Logroscino, 2005). A study in C57BL/6 mice daily treated with pQ (0.3 mg/Kg) or maneb (1 mg/Kg) or PQ + maneb from postnatal day 5–19 and then re-exposed as adults to PQ (10 mg/Kg) or maneb (30 mg/Kg) or PQ + maneb (twice a week/3 weeks) showed that dopaminergic cell loss and decreased dopamine levels were amplified by the adult re-challenge to the pesticides, suggesting that the developmental exposure to neurotoxins enhanced the adult susceptibility to a new toxic insult (Thiruchelvam et al., 2002). Similarly, prenatal exposure of pregnant C57BL/6J mice to PQ (0.3 mg/Kg) or maneb (1 mg/Kg) altered the development of the nigrostriatal system and enhanced its vulnerability to neurotoxins later in life, which could contribute with the development of PD during aging (Barlow et al., 2004).

Although there is no direct evidence linking early exposure to environmental pollutants and epigenetic changes with increased susceptibility to LOPD, there is a plausible association based on the following considerations: (1) epigenetic alterations have been observed in PD brains; (2) the exposure to environmental factors is associated with an increased risk of LOPD development and factors such as pesticides and metals can alter mechanisms of epigenetic regulation such as DNA methylation; and (3) early exposure to environmental pollutants might be associated with LOPD later in life. Further studies are needed to confirm this hypothesis in this promising research field to understand the mechanisms underlying the long-term effects of the environment on the PD development.

## Concluding Remarks

The emerging association between exposures to several toxic compounds with neurodegenerative diseases is of considerable public health importance, given the increasing dementia prevalence, the negative social and economic consequences of neurodegeneration-related disabilities, and the increasing environmental pollution in some geographic areas worldwide. Some of the epidemiological studies show not consistent results on getting significant estimates of hazard risk for AD or PD, mainly due to some limitations that include the difficulty on accurate diagnosis for AD or PD cases due to the lack of specific biomarkers, the deficiency to accurately assess chronic exposures, and/or the lack of inclusion of important confounding variables such as co-exposure to toxic compounds, genetic variants and lifestyle among others. Nevertheless, epidemiological studies along with experimental data have led to highlight the potential risk to develop these degenerative diseases due to the exposure to environmental pollutants such as metals, NPs and pesticides, among others. Interestingly, these pollutants show similar mechanisms of toxicity, which converge in a generalized mechanism based on the generation of oxidative stress that leads to common hallmarks of both neurodegenerative disorders. For example, the generation of oxidative stress by increasing the production of ROS and/or deregulating the antioxidant enzymes promotes the formation of protein aggregates such as Tau, Aβ or α-syn. This in turn overwhelms the degradation systems, and produces the activation of the glia inducing neuroinflammation, a process that *per se* increases the generation of further oxidative stress leading to a self-perpetuating cycle, and finally to neuronal loss of specific brain region such as the hippocampus and cerebral cortex in AD and substantia nigra in PD. The oxidative stress induced by these neurotoxicants activates/inhibits signaling pathways leading to augmented/diminished activity of enzymes that promote the accumulation of toxic materials in neural cells such as damaged/aberrant proteins, Aβ in AD or α–syn in PD and oxidative byproducts, or the oxidation of DNA that can alter genetic or epigenetic regulation. Furthermore, the link between early life exposure to environmental factors and the origin of neurodegenerative diseases is getting attention and can help to clarify the role of the environment on the development of these degenerative diseases. On the other hand, the lack of specific/differential biomarkers for AD or PD limits the early diagnosis and then the timely treatment. In this regard, specific circulating miRNAs have been associated with pathological processes such as AD and PD, therefore they are promising non-invasive biomarkers for these neurological diseases. Additionally, the identification of biomarkers to determine the past exposure to environmental pollutants is of vital importance for a better and opportune management of these diseases. Thus, as we have more knowledge of the risk from the exposure to environmental pollutants, more well-designed epidemiological studies (controlling for as many variables as possible and with high sample sizes) are necessary to improve the quality of life of elderly and to prevent the development of neurodegenerative diseases worldwide.

## Conflict of Interest Statement

The authors declare that the research was conducted in the absence of any commercial or financial relationships that could be construed as a potential conflict of interest.

## Abbreviations

Aβ: amyloid beta peptide
AICD: amyloid intracellular domain
AD: Alzheimer’s disease
Al: aluminum
AGE: advanced glycation end products
ApoE: apolipoprotein E
APP: amyloid precursor protein
As: arsenic
*α*-syn: alpha-synuclein
BACE1: beta-site APP cleaving enzyme 1
Cd: cadmium
CSF: cerebrospinal fluid
EOAD: early-onset Alzheimer’ disease
Hg: mercury
HR: hazard ratio
IDE: insulin-like degrading enzyme
LB: Lewy bodies
LOAD: late-onset Alzheimer’ disease
LRP1: low density lipoprotein receptor-related protein 1
Me-Hg: methyl mercury
Mn: manganese
MTs: microtubules
NEP: neprilysin
NFTs: neurofibrillary tangles
NPs: nanoparticles
OP: organophosphates
OCl: organochlorines
PHFs: paired helical filaments
Pb: lead
PD: Parkinson’s disease
PQ: paraquat
PS: presenilin
ROS: reactive oxygen species
RR: relative risk
sAPPβ: soluble APP beta
SNpc: substantia nigra pars compacta
SNpr: substantia nigra pars reticulate
Tau: tau protein
P-Tau: phosphorylated Tau
TCE: trichloroethylene
Zn: zinc.
